# Text mining methods for automated data extraction from health technology assessment reports of medicines using classical natural language processing and generative artificial intelligence

**DOI:** 10.1093/jamiaopen/ooag051

**Published:** 2026-04-27

**Authors:** Jan-Willem Versteeg, Marie L De Bruin, Maarten Schermer, Shiva Nadi Najafabadi, Modhurita Mitra, Christine Leopold, Aukje K Mantel-Teeuwisse, Wim G Goettsch, Lourens T Bloem

**Affiliations:** Division of Pharmacoepidemiology and Clinical Pharmacology, Utrecht Institute for Pharmaceutical Sciences, Utrecht University, Utrecht, The Netherlands; Division of Pharmacoepidemiology and Clinical Pharmacology, Utrecht Institute for Pharmaceutical Sciences, Utrecht University, Utrecht, The Netherlands; Research Engineering, Utrecht University, Utrecht, The Netherlands; Research Engineering, Utrecht University, Utrecht, The Netherlands; Research Engineering, Utrecht University, Utrecht, The Netherlands; Division of Pharmacoepidemiology and Clinical Pharmacology, Utrecht Institute for Pharmaceutical Sciences, Utrecht University, Utrecht, The Netherlands; Division of Pharmacoepidemiology and Clinical Pharmacology, Utrecht Institute for Pharmaceutical Sciences, Utrecht University, Utrecht, The Netherlands; Division of Pharmacoepidemiology and Clinical Pharmacology, Utrecht Institute for Pharmaceutical Sciences, Utrecht University, Utrecht, The Netherlands; Zorginstituut Nederland, Diemen, The Netherlands; Division of Pharmacoepidemiology and Clinical Pharmacology, Utrecht Institute for Pharmaceutical Sciences, Utrecht University, Utrecht, The Netherlands

**Keywords:** Automated data extraction, health technology assessment, generative AI, large language models, natural language processing

## Abstract

**Objective:**

This proof of concept for utilizing automatic data extraction methods to extract health technology assessment (HTA) attributes from HTA reports of medicines aimed to explore which attributes could be extracted and how accurately, using different data extraction methods. This enables easy access to insights into HTA recommendations for policymaking and policy-related research.

**Materials and Methods:**

In total, 14 relevant attributes (eg, assessment outcome or date) were identified for extraction using two classical natural language processing (NLP) methods (rule-based and classification models) and a generative AI method (large language model (LLM)-based, i.e., Claude 3 Opus). The performance of these techniques was compared using 50 HTA reports published by the National Institute for Health and Care Excellence (NICE, United Kingdom).

**Results:**

All three methods were able to extract certain attributes with high accuracy, with differences between the extraction methods and the type of attribute. The LLM-based extraction was the only method able to extract attributes on a medicine-indication combination level. The LLM-based extraction performed best (88–98% semantical accuracy for 12/14 attributes). Extraction of Outcome relative effectiveness analyses (REA) and Comparator was the most challenging and had the lowest accuracy (∼70% for the LLM-based extraction).

**Discussion & Conclusion:**

Automatic data extraction for relevant attributes from HTA reports is possible, but there is still room for improvement. LLM-based extraction outperformed the two NLP methods, but challenges regarding the use of commercial software and reproducibility remain. Future research should focus on expanding the system to other HTA organizations and further refining the LLM-based extraction.

## Background and significance

Health technology assessment (HTA) is a multidisciplinary process that is used to determine the value of health technologies at different points in their lifecycle to facilitate evidence-based decision-making regarding the reimbursement of the health technology.^1^ As HTA often supports national or regional policy making, differences in their execution exist between HTA organizations in the performed analyses, which stem from differences in methods and preferred evidence used in these analyses.^2^^,^^3^ To better understand these differences, comparative HTA research is performed. Past comparative HTA research has focused on comparing differences in, for instance, methods of data analysis, evidence assessed, and decisions or recommendations made.^3–6^ By understanding and evaluating these differences and informing improvements for decision-making, this research is an important factor in keeping healthcare equitable, affordable, and accessible in the future.

Data attributes that are used in comparative HTA research (such as assessment outcome and assessment date) often originate from publicly available HTA reports. Large repositories or databases that include these attributes are often incomplete, such as the HTA community-driven INAHTA database, or are not freely accessible, such as the commercial NAVLIN database.^7^^,^^8^ This results in the need for manual data collection by reading hundreds, if not thousands, of reports to extract the relevant attributes. A study into human errors in literature reviews has shown that this process is incredibly time-consuming and results in human errors of 10.78% (95% CI: 7.43% to 14.09%) in abstract screening.^9^ Consequently, the resources needed for manual data extraction and the data quality that can be reached limit the number and type of research questions that can be answered, and the reproducibility, updateability, and generalizability of the findings.

A potential solution is to automate the data extraction using text retrieval and text mining methods. These methods can be used to extract data from both structured and unstructured text.^10^^,^^11^ Automated data extraction methods range from classical natural language processing (NLP) (rule-based extraction and machine learning classification) to modern generative artificial intelligence (AI) approaches using large language models (LLMs). These methods have proven successful across diverse fields, including innovation sciences, healthcare, law, and construction.^12–16^

Text mining and other machine learning and AI methods are already being used in or discussed for some core HTA processes, such as evidence synthesis, evidence generation, or cost-effectiveness analysis.^17^^,^^18^ However, to our knowledge, no research into or use cases of these methods for automatically extracting data from HTA reports to support comparative HTA research exists. A possible reason for this is the unstructured and ambiguous nature of these documents, with large differences in document structure and wording both within and between HTA organizations.

## Objective

Therefore, this study aimed to explore which attributes could be extracted from HTA reports and how accurately they could be extracted, using different data extraction methods, including classical NLP-based text retrieval and text mining methods, and new LLM-based text retrieval and text mining methods.

## Material and methods

### Study design

This proof-of-concept study of automated data extraction from unstructured text in HTA reports was based on reports from one HTA organization, the National Institute for Health and Care Excellence (NICE) in the United Kingdom. NICE was chosen because they perform extensive HTA, including cost-effectiveness assessments (CEA), and publish their reports publicly in English on their website. We developed and assessed the performance of classical NLP-based text retrieval and text mining methods, and new LLM-based text retrieval and text mining methods, comparing them to manual data extraction, in extracting a set of research-relevant attributes from HTA reports.

### Attributes of interest

Fourteen data attributes, often used in comparative HTA research, were identified by experts with domain knowledge on HTA and comparative HTA research. Attributes ranging from unambiguous attributes, such as Assessment date, to more ambiguous (eg, Outcome of the relative effectiveness assessment (REA)) or less structurally reported (eg, Comparator) attributes were included. A list of these 14 attributes and their meaning can be found in Appendix 1, available as supplementary data at [*JAMIA Open*] online.

### Construction of the dataset and text pre-processing

HTA reports were obtained in PDF format through web scraping of NICE’s guidance repository on 19-01-2024 using the selection criteria “Technology appraisal guidance,” resulting in a total of 734 documents.^19^ Afterwards, the PDFs were converted to .txt format for to be used by the extraction methods. A test set, consisting of a randomly selected set of 50 reports, was created. The manual data extraction of these 50 reports identified a total of 60 medicine-indication combinations assessed in these reports. From these, our 14 attributes of interest were manually extracted to serve as the gold standard to compare the performance of the three text-mining approaches with. Manual extraction was performed by JV, and in cases where the correct value to extract was uncertain, it was discussed with LB. The documents included in the gold standard were not used for the development of any of the data extraction methods.

### Data extraction methods

To extract the attributes from the HTA reports, three different methods were developed in collaboration between research engineers and HTA researchers. A high-level description of the development phase of the text retrieval and text mining methods can be found in Box 1.

#### Rule-based natural language processing (NLP-R)

NLP-R extraction uses predefined rules to identify and extract information from unstructured text. This approach relies on pattern matching, part-of-text tagging, and other language-based techniques to locate and extract relevant data.

#### Classification model-based natural language processing (NLP-CM)

NLP-CM extraction utilizes machine learning algorithms to learn patterns and features from pre-labeled data, thereby training a model to predict and extract information from new, unseen text. Our analysis included the following classification models: Gradient Boosting, Support Vector Machine, Logistic Regression, Random Forest, Naive Bayes, and BERT(Bidirectional Encoder Representations from Transformers). The first five are classical machine learning models, widely used in NLP tasks for their simplicity, interpretability, and effectiveness with structured text representations. They often perform well for classification tasks.^20^ BERT, in contrast, is a deep learning model that leverages transformer-based architecture to capture bidirectional context, achieving state-of-the-art results across a wide range of NLP benchmarks.^21^

#### LLM-based

LLM-based extraction uses an LLM trained on large amounts of data to extract relevant information from text in an open-ended, contextual manner. These models can understand and interpret text at a deeper level, going beyond simple pattern matching to capture nuanced semantics and relationships. For our study, we used Claude 3 Opus^®^, developed by Anthropic.^22^

Box 1 Development of the data extraction methods

**NLP-R** 

The initial set of rules was developed by modeling the steps a human would take to extract the data manually. These rules were then refined iteratively based on the results. After each iteration, error analysis was conducted on the outputs, followed by implementing solutions to address identified issues. Specific points of improvement worth mentioning were the addition of a dictionary of brand names and INNs obtained from the Drugbank.com API and the development of rules focused on specific sections of the document instead of the entire document.

**NLP-CM** 

We started with the attribute final recommendation to optimize the model and training data. The first training set we used consisted of 200 manually labelled documents following labelling rules used in earlier comparative HTA research, available in Appendix 2, available as supplementary data at [*JAMIA Open*] online.^23^ There were many more positive than negative outcomes in the training set, which made the set too imbalanced to train the models. To overcome this, the imbalance in the training set/model was corrected by applying oversampling techniques. Thereafter, we added an alternative training method that only trained on a specific document section for the Final recommendation, and we increased the previous training set to 300 labelled documents. In total, the final training set consisted of 247 annotated documents, as 53/300 documents were terminated assessments and could not be labelled. With these improvements, the classification models reached F-1 scores above 0.90 for the Final recommendation. We then used the larger training set for the attributes Outcome REA and Outcome CEA. For Outcome CEA, we also defined a specific document section, but for Outcome REA, this was impossible.

**LLM-based** 

We started with a trial-and-error approach to find the right questions to ask the LLM. Thereafter, we used different prompt engineering techniques to increase performance and reproducibility. The most important improvement was limiting the answers the LLM could give to certain options. Another important improvement was the transition from a question-based prompt to our final schematic prompt, Appendix 3, available as supplementary data at [*JAMIA Open*] online. This schematic prompt was based on the NICE document structure and allowed us to extract attributes on a medicine-indication combination level by allowing multiple medicines and indications of interest for each report. During the development phase, we tested different LLMs from OpenAI and Anthropic. Claude 3 Opus performed best for our use case and was used for further development and final application.

*Box 1: information on the development of the three extraction methods. Abbreviations: application programming interface (API), cost-effectiveness assessment (CEA), health technology assessment (HTA), international non-proprietary name (INN), large language model (LLM), natural language processing classification models-based (NLP-CM), natural language processing rule-based (NLP-R), relative effectiveness assessment (REA).*

### Attributes per extraction method

Not all attributes were extracted using all three data extraction methods, as NLP-R and NLP-CM were used complementarily. NLP-CM extraction can only be used for attributes that can be classified, and as such, was only used for the attributes Final recommendation, Outcome REA, and Outcome CEA. Because we were unable to formulate extraction rules for these three attributes, NLP-R extraction was deemed unfit to extract these attributes. Lastly, clinical restrictions were only extractable using LLM-based extraction, as it is not a classifiable datapoint, and no rule could be developed for NLP-R extraction. The 14 attributes and the data extraction methods used to extract them from the reports are described in Table 1.

**Table 1.** Attributes and the data extraction methods that were used to extract them from the HTA reports.

Attribute	Structurally available/unavailable or classifiable	NLP-R	NLP-CM	LLM
Internal identifier	Available	**x**		**x**
HTA ID	Available	**x**		**x**
Brand name	Available	**x**		**x**
INN	Available	**x**		**x**
MAH	Available	**x**		**x**
Assessment type	Available	**x**		**x**
Assessment date	Available	**x**		**x**
Indication	Available	**x**		**x**
Final recommendation	Classifiable		**x**	**x**
Comparator	Unavailable	**x**		**x**
Outcome REA	Classifiable		**x**	**x**
Outcome CEA	Classifiable		**x**	**x**
MEA	Unavailable	**x**		**x**
Clinical restrictions	Unavailable			**x**

A distinction is made between structurally available attributes, less structurally available attributes, and classifiable attributes.

*Abbreviations: cost-effectiveness assessment (CEA), health technology assessment organization name (HTA ID), international non-proprietary name (INN), large language model (LLM), managed entry agreement (MEA), marketing authorization holder (MAH), natural language processing classification models-based (NLP-CM), natural language processing rule-based (NLP-R), relative effectiveness assessment (REA)*.

### Analysis

Due to inherent differences between the extraction methods, separate analyses were performed for each of the three data extraction methods. For the NLP-R and NLP-CM extractions, we analyzed the results at the document level, as extraction at the medicine-indication combination level was found to be impossible during the development of the methods. For the LLM-based extraction, we analyzed attributes at the medicine-indication combination level and performed an additional analysis on reproducibility.

#### NLP-R

NLP-R extraction was assessed for textual accuracy, i.e., an exact textual match between NLP-R extraction and the gold standard. The attributes that were not a textual match were assessed on useful accuracy, i.e., a broader selection of text extracted, which contains the right value for the attribute, but also additional text (examples can be found in Appendix 4, available as supplementary data at [*JAMIA Open*] online). Both were expressed in percentages of the total set of values for each attribute.

#### NLP-CM

For NLP-CM extraction, we annotated the 50 gold standard documents with a positive or negative value for Final recommendation, Outcome REA, and Outcome CEA based on the same set of classification rules used for creating the training set (Appendix 3, available as supplementary data at [*JAMIA Open*] online). The performance of the different classification models was then evaluated on the annotated gold standard, using confusion matrices, F1-scores, and area under the curve (AUC) metrics to compare the models.^24^

#### LLM-based

For the LLM-based extraction, we first discarded medicine-indication combinations that the LLM-based extraction identified but were not identified in our gold standard. We classified those records as hallucinations if we could not identify any reason or source for the discrepancy in the respective document and as misclassifications if we could identify a reason or source for the discrepancy. For the remaining 60 medicine-indication combinations of our gold standard, we calculated the textual accuracy, meaning an exact textual match with our gold standard. In cases without a textual match, we assessed the semantic accuracy, meaning not the same wording but the same semantic meaning. As reproducibility can be an issue with using LLMs, we analyzed the reproducibility of our extraction by running the entire extraction a second time and analyzing the differences between the two extractions.

### Interpretation

We defined a percentage of 90% as research relevant because we consider this percentage to be sufficient to inform comparative HTA research, taking into account inter-rater agreements found in comparative HTA research and the fact that manual data extraction/labelling is prone to errors of over 10%.^9^^,^^25^^,^^26^

## Results

Because different analyses were performed for each of the developed extraction methods, results for each method will be presented separately.

### NLP-R extraction

**Figure 1.**
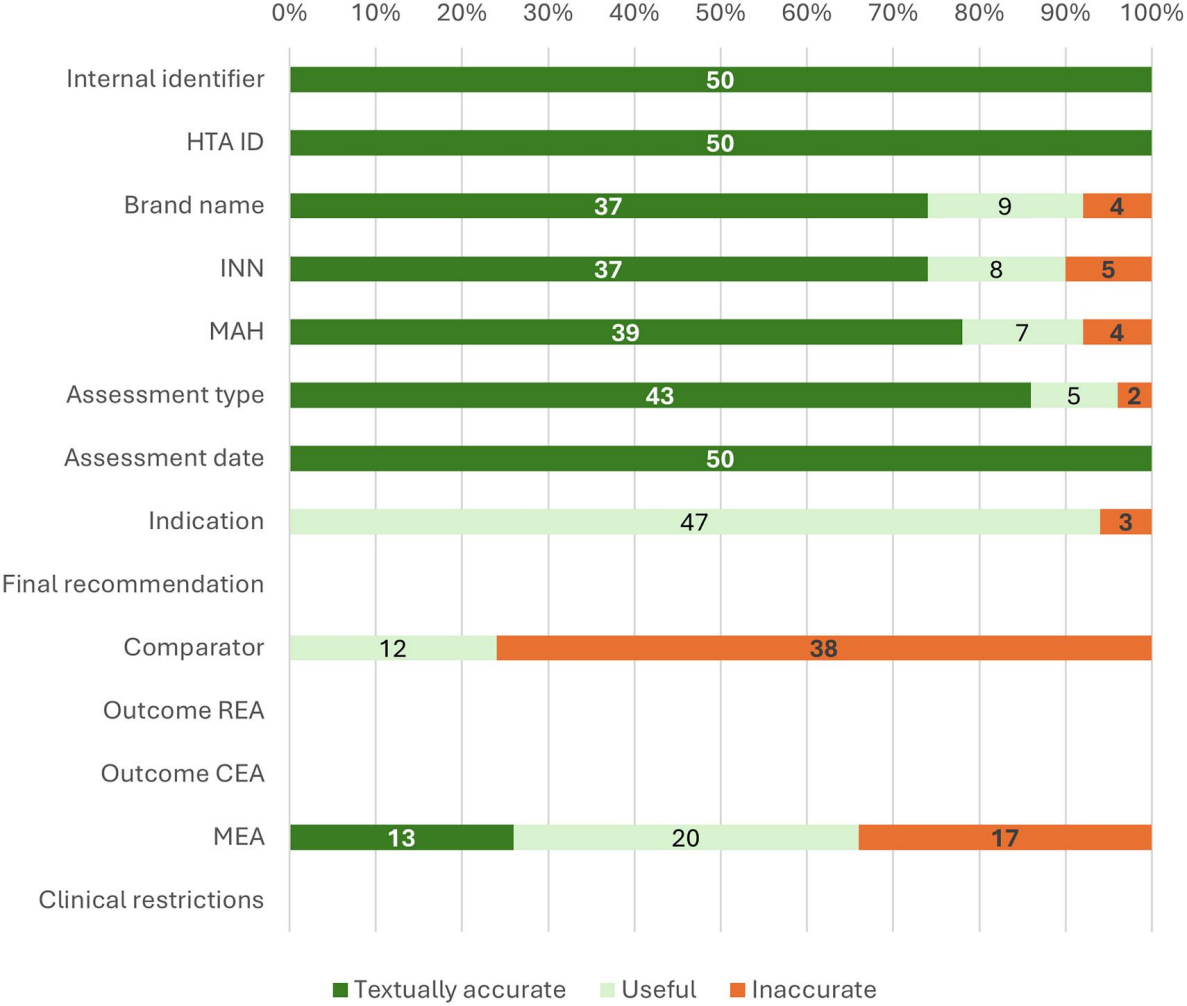
Results of the NLP-R extraction displaying the number of attributes textually accurate, useful, and inaccurate extracted on a document level. Attributes labelled “inaccurate” contain both incorrect and missing extractions. More details on incorrect versus missing extractions can be found in Appendix 5, available as supplementary data at [JAMIA Open] online. Abbreviations: cost-effectiveness assessment (CEA), health technology assessment organization name (HTA ID), international non-proprietary name (INN), managed entry agreement (MEA), marketing authorization holder (MAH), relative effectiveness assessment (REA).

NLP-R extraction performed well on 5/10 attributes (50%) for exact accuracy, or 8/10 attributes (80%) when including useful results containing relevant information, see Figure 1.

#### Structurally available attributes

For structurally placed attributes, NLP-R achieved high textual accuracies. Internal identifier, HTA ID, and Assessment date all scored 100% accuracy. Assessment type classification achieved 86% accuracy, with lower scores primarily due to reassessment reports where the system extracted “this TA replaces…” text rather than identifying the document as a reassessment itself.

INN and Brand name extraction showed lower textual but high usefulness scores (90% and 92% respectively), as redundant words were sometimes extracted alongside the target information. Most INN errors involved extracting additional INNs mentioned for earlier treatment lines. Brand name errors included returning INNs when no brand name was available or missing multiple brand names in documents assessing multiple medicines.

Indication extraction scored well on usefulness (94%) but 0% on textual accuracy, as it was consistently extracted from concluding paragraphs that contained the indication, but were extracted with other redundant text in the paragraph.

#### Less structurally available attributes

For attributes lacking consistent structural placement, performance was notably lower. MEA extraction achieved only 26% accuracy and 66% usefulness, with the most common error being failure to detect existing MEAs (16/17 cases). Comparator extraction performed worst due to heterogeneous reporting across NICE reports over time, with the primary error being failure to identify any comparator when one existed (30/38 errors). An overview of incorrect versus missing extractions for the NLP-R extractions can be found in Appendix 5, available as supplementary data at [*JAMIA Open*] online.

### NLP classification model-based

**Figure 2.**
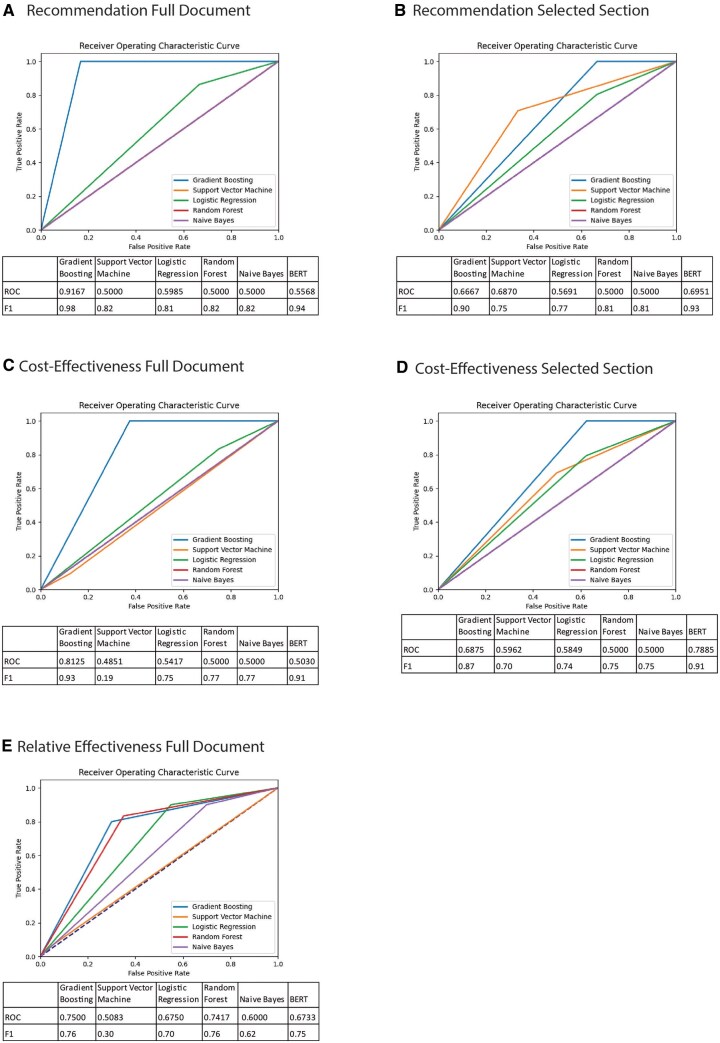
Results of the classification models for Final recommendation (A and B), Outcome CEA (C and D), and Outcome REA (E). A, C, and E were trained on the full document, whereas B and D were trained on a specific section of the document, as shown in Box 1.

Figure 2 shows the results of the classification models. When looking at the obtained F-1 scores, a combination of precision and recall of the model, we see that for the parameter Final reimbursement recommendation, the best model was the gradient boosting model on the full document with an F-1 score of 0.98. For Outcome CEA, the best model was the gradient boosting model on the full document with an F-1 score of 0.93. For Outcome REA, the best models were gradient boosting and random forest, with an F-1 score of 0.76. The confusion matrices of these models are shown in Tables 2a–d.

**Table 2a.** Confusion matrix of the gradient boosting classification model for the Final reimbursement recommendation, trained and applied to the full document.

Final reimbursement recommendation	Predicted Negative	Predicted Positive
**True Positive**	5	1
**True Positive**	0	44

**Table 2b.** Confusion matrix of the gradient boosting classification model for Outcome Cost Effectiveness Analysis, trained and applied to the full document.

Cost-effectiveness	Predicted Negative	Predicted Positive
**True Negative**	5	3
**True Positive**	0	42

**Table 2c.** Confusion matrix of the gradient boosting classification model for Outcome Relative Effectiveness Analysis, trained and applied to the full document.

Relative effectiveness Gradient Boosting	Predicted Negative	Predicted Positive
**True Negative**	14	6
**True Positive**	6	24

**Table 2d.** Confusion matrix of the random forest classification model for Outcome Relative Effectiveness Analysis, trained and applied to the full document.

Relative effectiveness Random Forest	Predicted Negative	Predicted Positive
**True Negative**	13	7
**True Positive**	5	25

Based on the test set of 50 reports, there was a clear majority class (positive) with 44 and 42 positive outcomes, respectively, for the final reimbursement recommendation and Outcome CEA. The confusion matrices show that the models were flawless in predicting these values. For the minority (negative) class, the models made some misclassifications, 5/6 (83%) for Final recommendation were correctly classified, and 5/8 (63%) for Outcome CEA. The test set for relative effectiveness was divided more equally between positive (30) and negative outcomes (20). For the 30 positive values for Outcome REA gradient boosting classified 24/30 (80%) correctly, and random forest 25/30 (83%), for the 20 negative values, gradient boosting classified 14/20 (70%) correctly, and random forest 13/20 (65%). Generally, the classification models performed worse in predicting Outcome REA. For Outcome REA, both the gradient boosting and the random forest model made mistakes in both the majority and the minority class.

### Gen-AI LLM

**Figure 3.**
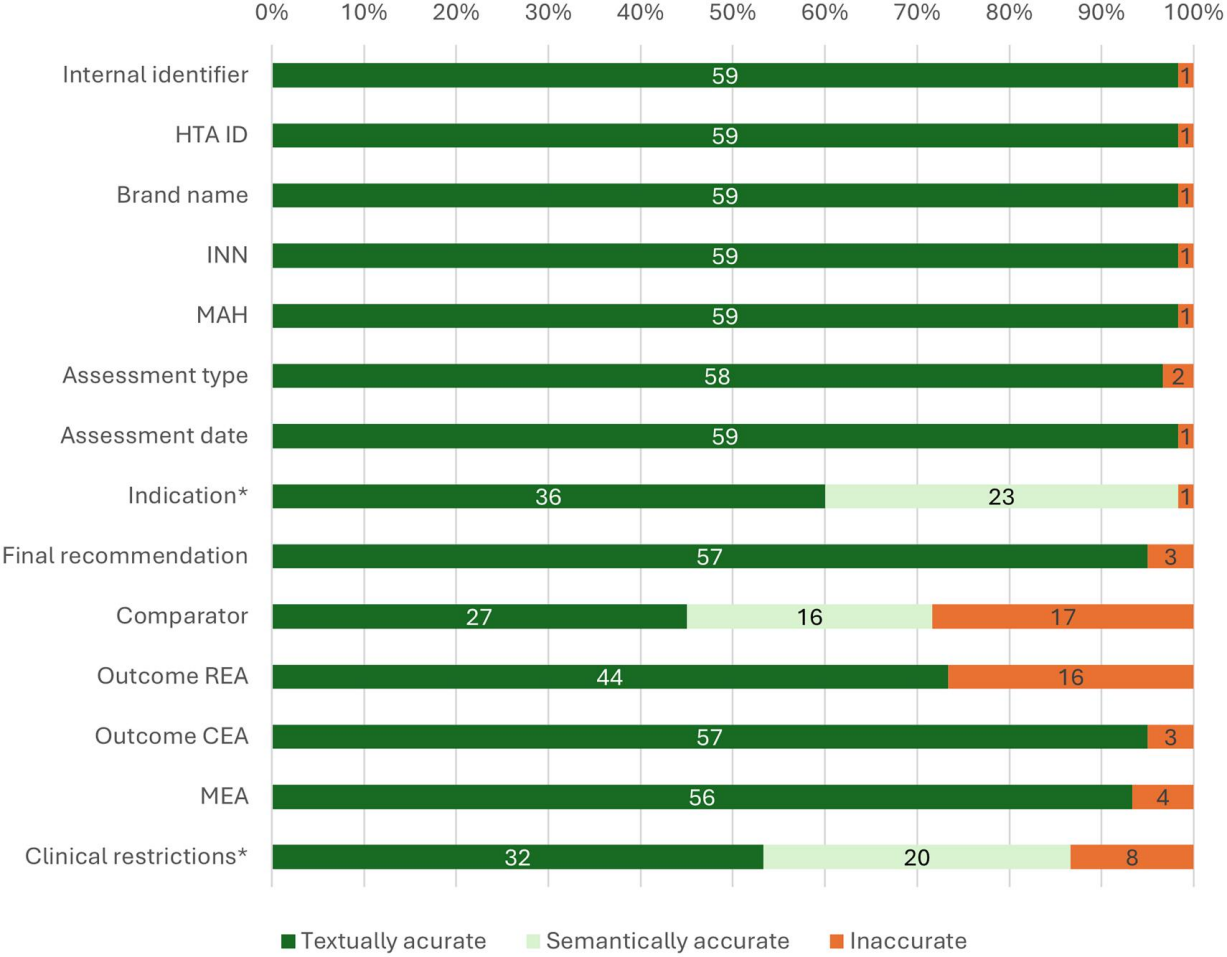
Extraction accuracy for the LLM-based extraction. Accuracy is calculated on a textual level and a semantic level. Textual accuracy shows an exact textual match, and semantic accuracy shows a non-exact textual match that semantically means the same. *Indication-specific and clinical restrictions were often extracted and combined by the LLM. For these attributes, a correct extraction of one of the two attributes was deemed semantically accurate. Abbreviations: cost-effectiveness assessment (CEA), health technology assessment organization name (HTA ID), international non-proprietary name (INN), managed entry agreement (MEA), marketing authorization holder (MAH), relative effectiveness assessment (REA).

Figure 3 shows the results of the analysis of the LLM-based extraction. The LLM identified three medicine-indication combinations that were not identified in the gold standard. These three medicine-indication combinations were deemed misclassifications, as there was a clear source in the documents from which these three medicine-indication combinations originated, which explained why they could have been identified and were therefore not hallucinations. These were excluded from the result table as they did not reflect medicine-indication combinations assessed in the documents. For the 60 medicine-indication combinations identified in the gold standard, the LLM-based extraction identified 59, resulting in at least one inaccurate extraction for all attributes.

#### Structurally available attributes

For the structurally available attributes Internal identifier, HTA ID, Brand name, INN, MAH, and Assessment date, the missing medicine-indication combination was the only error made.

Indication and Clinical restrictions had lower textual accuracies because of an interaction between the two. Identifying what is an indication and what is a clinical restriction is difficult, as NICE reports document this inconsistently. Because of this, clinical restrictions were sometimes added to the indication attribute in the LLM-based extraction, leading to formally incorrect extractions for both Indication and Clinical restrictions. We considered these errors as semantically accurate when they contained the correct information on one or both attributes. In total, 13 such errors were found in Indication and one additional error in Clinical restrictions. The additional error in Clinical restrictions comes from an extraction where part of the indication was extracted both in the Indication and the Clinical restrictions, giving an error for Clinical restrictions but not for Indication.

#### Analysis outcome classification

When classifying the Final recommendation and the Outcome CEA attributes, the LLM was highly accurate (95%). Classifying Outcome REA was notably harder (73%). This lower accuracy was mostly because the LLM-based extraction classified REAs as positive that were classified as negative in the gold standard, and the Outcome REA was assessed as too uncertain in the gold standard.

#### Less structurally available attributes

In contrast to NLP-R extraction, the LLM-based extraction was highly accurate in identifying and extracting MEAs. The accuracy of the Comparator was lower, with 72% semantic accuracy. Most errors occurred when the LLM-based extraction identified more comparators than the gold standard. In these cases, the reports identified more comparators that were not deemed the most relevant comparators by NICE, which were then wrongfully extracted by the LLM. Other errors were made when the Comparator extracted by the LLM was “standard of care”, where the gold standard contained more detailed information on what this standard of care entailed (eg, chemotherapy or plasma exchange and immunosuppressant medicines).

#### Reproducibility results

To test the reproducibility of the LLM-based extraction results, the model was run again at a different moment using the same input files and extraction schema. In total, 19 differences were found for 11 reports (2,26% of all 840 extracted attribute values). Of these 19 differences, five were irrelevant as they only concerned a changed order of words or a difference in punctuation. Of the 14 remaining differences (1,67% of all 840 extracted attributes values) that yielded a semantically different result, four concerned the Comparator, three concerned medicine-indication combinations (two initially identified medicine-indication misclassifications were not identified anymore, but one other misclassification was identified), two concerned Clinical restrictions and Indication and one each was found in Final recommendation, Outcome REA, and Outcome CEA.

## Discussion

### Summary of results

Comparative HTA research is used for evaluating, improving, and updating HTA policies, processes, and methods. Currently, comparative HTA research is heavily dependent on manual data extraction from HTA assessment reports by researchers. With this proof-of-concept study, we aimed to explore which attributes could be extracted from HTA reports and how accurately they could be extracted, using different data extraction methods, including classical NLP-based text retrieval and text mining methods, and new LLM-based text retrieval and text mining methods. When comparing the three extraction methods, the LLM-based extraction consistently outperformed both NLP-R extraction and NLP-CM extraction in terms of accuracy. The LLM-based method was also easier to develop and had a lower development time compared to the NLP methods. It was able to extract most attributes with high accuracy (88–98% for 12 out of 14 attributes), required limited programming knowledge, and proved more adaptable across diverse HTA report formats. NLP-R extraction performed well for attributes that were structurally placed and consistently formatted, such as the TA number and Assessment date, but struggled with more textually nuanced or variably located information, including Comparators and MEAs. In contrast, NLP-CM was specifically applied to classify the attributes that related to key outcomes (Final recommendation, Outcome REA, and Outcome CEA), achieving high performance for binary classification tasks with balanced data. However, it was limited by the small and imbalanced dataset, as well as the inability to process multiple medicine-indication combination assessments within a single document.

### Level of extraction

An important consideration across all methods was the level at which extraction could be performed. Both NLP-R and NLP-CM methods faced limitations when HTA reports contained multiple medicine-indication combinations within a single document. NLP-R extraction was unable to always extract multiple medicines and indications from one document and could not link different medicines to specific indications or comparators, limiting its use for assessments containing multiple medicine-indication combinations. Similarly, NLP-CM extraction could only make one classification per document and was not able to return multiple different outcomes when an HTA report contained assessments of multiple medicine-indication combinations. The LLM-based extraction was able to extract multiple medicine-indication combinations from one document, but also encountered challenges at this level, creating three new medicine-indication combinations compared to the gold standard due to misclassification of text within the reports.

### Structurally placed and consistently formatted attributes

For attributes that were more structurally available and consistently placed in the reports, such as Internal identifier and Assessment date, both NLP-R and LLM-based methods performed well. NLP-R extraction proved to be a feasible approach for data extraction when dealing with these structurally formatted attributes. However, even for some structurally available attributes, NLP-R extraction faced challenges in limiting extraction to exactly the required text segment. In those cases, rules were created to extract larger chunks of text around the required information, resulting in textually inaccurate but useful extractions that could speed up future research but would require further processing for direct research use. This requirement for extensive rule creation represented a key challenge specific to the NLP-R extraction.

### Analysis outcome classification

Both NLP-CM and LLM-based methods were applied to classify key HTA outcomes, with varying degrees of success. Final recommendation and outcome CEA could be classified very well using both approaches, with NLP-CM extraction achieving F1 scores of 0.98 and 0.93, respectively, and LLM-based extraction achieving accuracies of 88-98%. However, both methods performed less optimally for outcome REA classification. NLP-CM extraction resulted in a lower F1 score of 0.76 for REA outcomes, reflecting more false positive and false negative classifications. This was likely caused by NICE making fewer hard conclusions in their text about REA outcomes compared to final recommendations and CEA outcomes, which makes it harder to classify as either positive or negative. The LLM-based extraction also resulted in a lower accuracy (72-73%) for outcome REA extraction. Notably, the LLM-based approach offered greater granularity in classification, including 3 Outcome REA categories, as NLP-CM extraction worked best with binary classification due to the small and highly unbalanced training set. The NLP-CM extraction was particularly challenged by the limited availability of HTA documents for training, resulting in small and unbalanced datasets with more positive than negative examples. Despite using BERT, a state-of-the-art deep learning model with transformer-based architectures, classical models were not outperformed in this task, possibly due to factors such as data size, domain specificity, or the nature of the classification problem.

### Less structurally available attributes

For attributes that were more textually described, less consistently formatted, and located in different places throughout documents, such as Comparator and MEA, the methods showed different performance levels. NLP-R extraction performed worse for these data points that had to be identified in different places across different documents. In contrast, the LLM-based approach struggled less with most of these attributes, which was expected given the context understanding capabilities of LLMs compared to NLP-R methods. However, even the LLM-based extraction showed limitations, with comparator extraction achieving lower accuracy (72-73%), likely due to the way this information is stated in NICE assessment reports. The LLM-based approach, while showing superior overall performance, carried inherent risks including potential hallucinations. Although efforts were made to limit this by including options for the LLM to return no value when none was found, and no completely fabricated attributes or medicine-indication combinations were extracted, misclassifications did occur.

### Strengths and limitations

A strength of our study is that this is the first to study the possibility of automated data extraction from HTA reports. In addition, our study evaluated three different data extraction methods to assess which one would perform best in our use case.

A limitation of our study is the external validity of our findings. We only used NICE assessment reports and extracted 14 high-level data attributes; as such, no definitive conclusions can be made on how automated data extraction using these three methods would perform for other organizations, data attributes, and in other languages.

A factor that can be both a strength and a limitation is the use of a commercial state-of-the-art LLM, Claude 3 Opus. As the field is moving very fast, updates to the model or new models would warrant continuous evaluation of the LLM-based extraction, but could potentially also further improve the usefulness and accuracy.

### Future outlook

Due to its higher accuracy, the ability to extract multiple medicine-indication combinations from a single report, lower development times, and minimal structural dependence, automatic data extraction using our developed LLM-based extraction is the most feasible option for current use and further development. Data attributes with an accuracy of 90% or higher (11/14 attributes, 1 attribute at 88%) can already be used for comparative HTA research. Further refinement should focus on reducing the misclassifications and improving the accuracy of extracting the outcome of REA and the comparator.

To address hallucinations and misclassifications, future efforts could study incorporating either multiple extraction runs with consistency checks or a researcher-in-the-loop approach, or both.^27^ A researcher-in-the-loop approach could involve reviewing flagged inconsistencies, validating low-confidence extractions, or spot-checking random samples. Such hybrid approaches would combine automated efficiency with human expertise to guarantee the quality of the extracted data.

The extracted data from NICE reports is a first step toward creating a database with relevant attributes extracted from HTA reports that can be updated easily and regularly. To further advance towards such a database, future research and development will focus on evaluating the transferability of the LLM-based data extraction method to HTA reports from HTA organizations other than NICE, expanding to other, less commonly used languages, and more complex document structures. Collaboration with HTA organizations in the development of country-specific LLM extraction schemas could increase development speed and quality through data and knowledge availability, and increase the uptake of our automated data extraction in practice.

Lastly, this proof of concept was performed using a commercially available model by Anthropic. Future research should also evaluate open source LLMs.

## Conclusion

Our study demonstrates that automated data extraction from unstructured HTA reports is both feasible and accurate enough for comparative HTA research. Extraction accuracy highly depended on attribute types, but among the three methods evaluated, the LLM-based extraction outperformed rule-based and classification models by extracting 12 out of 14 attributes with high accuracy, feasible development time, and ability to extract information on multiple medicine-indication combinations in a single document. Future efforts should focus on refining the LLM-based extraction, exploring researcher-in-the-loop systems, adding more data attributes, and transferring the system to other HTA organizations.

## Supplementary Material

ooag051_Supplementary_Data

## Data Availability

The source data for this study (NICE technical appraisals) are publicly available online on the NICE website. The data and code used in this study are publicly available on GitHub at: https://github.com/UtrechtUniversity/health-technology-assessment/tree/main/hta/LLM.
