# Supplementary material for: Text mining methods for automated data extraction from health technology assessment reports of medicines using classical natural language processing and generative artificial intelligence
Source: JAMIA Open. 2026 Apr 27;9(2):ooag051. doi: 10.1093/jamiaopen/ooag051 (PMC13120885; doi:10.1093/jamiaopen/ooag051)
Supplement: ooag051_Supplementary_Data [file ooag051_supplementary_data.zip › 2025.10.09 Supplementary file - appendices 1-5.docx]

**Appendix 1: Table of extracted datapoints and their meaning**

| **Attribute name** | **Attribute type** | **Attribute description** |
| --- | --- | --- |
| Internal identifier | Free Text | Internal organizational identifier. Often a number or a recurrent code. |
| HTA ID | Abbreviation | An abbreviation indicating which HTA organization is the assessor in a specific document. |
| Brand name | Free Text | The brand name of an assessed medicine in a report. |
| INN | Free Text | International nonproprietary name: the nonproprietary name of an assessed medicine in a report, also known as a generic name. |
| MAH | Free Text | Marketing authorization holder: the company/organization holding the marketing authorization at the regulatory level for the medicine assessed in the report. |
| Assessment type | Category | Type of assessment in the report. This can be an initial assessment, a reassessment, or a broadening of the indication. |
| Assessment date | Date | The date on which the assessment is published |
| Indication | Free Text | The specific indication for which the reimbursement of the medicine is assessed. |
| Final recommendation | Categorical | The final reimbursement recommendation (or decision, depending on the HTA organization's mandates). This can be positive or negative. |
| Comparator | Free Text | The current standard of care with which the assessed medicine is compared. Collected as INN, not as brand name, which is typically not provided. |
| Outcome REA | Categorical | The outcome of the relative effectiveness assessment. This can be positive, negative for NLP-CM and positive, negative, or equal for LLM-based. |
| Outcome CEA | Categorical | The outcome of the cost-effectiveness analysis. This can be positive or negative. |
| MEA | Categorical | Whether a managed entry agreement is proposed, and whether this is a financial-based or outcome-based agreement. |
| Clinical restrictions | Free Text | Any further clinical restrictions applicable to the final recommendation that are not captured in the specific indication. |

Appendix Table 1: Overview of extracted attributes and an explanation of their meaning. Abbreviations: cost-effectiveness assessment (CEA), health technology assessment organization name (HTA ID), international non-proprietary name (INN), managed entry agreement (MEA), marketing authorization holder (MAH), relative effectiveness assessment (REA).

**Appendix 2: Classification rules used for NLP-CM approach**

| **Final reimbursement positive** | **Final reimbursement negative** | **Added benefit** | **No added benefit** | **Cost effective** | **Not cost-effective** |
| --- | --- | --- | --- | --- | --- |
| Recommended for routine use | not recommended for routine use | All levels of added benefit | Equal benefit | ICER below threshold | ICER above threshold |
| Restricted | only in research |  | non-quantifiable benefit |  | Unclear because of limited data (cancer drug fund) |
| Managed entry | In the cancer drug fund |  | Lesser benefit |  |  |
|  | One of the assessed medicine-indication combinations is negative |  | Uncertain benefit >> explicit statement that the added benefit is uncertain. NOT that there is some uncertainty. |  | One of the assessed medicine-indication combinations is negative |
|  |  |  | the only benefit is user-friendliness |  |  |
|  |  |  | One of the assessed medicine-indication combinations is negative |  |  |

Appendix Table 2: Overview of the classification rules used to create the training set for the NLP-CM approach. Rules are based on classification rules used in earlier comparative HTA research. Abbreviations: incremental cost-effectiveness ratio (ICER)

**Appendix 3: Final extraction schema and full prompt YAML used for the LLM-based extraction.**

schema {}

├── hta_id

├── treatment_type

├── assessment_type

├── assessment_date

├── internal_identifier

└── indications [{}]

├── indication_name

└── technologies [{}]

├── is_combination_therapy

├── inn_combination_therapy

├── components [{}]

│ ├── inn_component

│ ├── brand_names [{}]

│ │ ├── brand_name

│ │ └── ema_mah

│ └── individual_comparators []

├── administered_with

├── combination_comparators []

├── outcome_rea

├── outcome_cea

├── final_recommendation

├── managed_entry_agreements

└── clinical_restrictions

**YAML version**

$schema: "http://json-schema.org/draft-07/schema#"

type: object

properties:

hta_id:

type: [string, "null"]

enum: ["NICE (UK)", "HAS (France)", "ZIN (Netherlands)", null]

description: "HTA organization performing the assessment"

treatment_type:

type: [string, "null"]

enum: [medicine, device, therapy, unknown, null]

description: "Specifies whether a medicine (drug), device, or therapy is being assessed"

assessment_type:

type: [string, "null"]

enum: ["initial assessment", reassessment, "indication broadening", "non-submission", unknown, null]

description: "Type of assessment being performed"

assessment_date:

type: [string, "null"]

pattern: "^\\d{4}-\\d{2}-\\d{2}$"

description: "Date of publication of assessment in YYYY-MM-DD format"

internal_identifier:

type: [string, "null"]

description: "Document's internal identifier (document ID), if available"

indications:

type: array

items:

type: object

properties:

indication_name:

type: [string, "null"]

description: "Name of indication for which the drug is assessed. The indication should be described in detail, including demographic (age, disease stage, etc.) for whom the treatment is intended, if this information is available."

technologies:

type: array

items:

type: object

properties:

is_combination_therapy:

type: boolean

description: "Indicates if this entry represents a combination of drugs assessed together"

inn_combination_therapy:

type: [string, "null"]

description: "International Non-Proprietary name of drug or combination, if this is a combination therapy"

components:

type: array

items:

type: object

properties:

inn_component:

type: [string, "null"]

description: "International Non-Proprietary name of individual drug component"

brand_names:

type: array

items:

type: object

properties:

brand_name:

type: [string, "null"]

description: "Brand name of drug"

ema_mah:

type: [string, "null"]

description: "Name of EMA Marketing Authority Holder"

required: [brand_name, ema_mah]

description: "Brand names associated with this drug component"

individual_comparators:

type: array

items:

type: [string, "null"]

oneOf:

- enum: ["standard of care", "best supportive care", placebo, "no treatment", "conventional care", "palliative care", null]

- type: string

description: "Name of drug(s) that this component drug is assessed against"

pattern: "^[a-zA-Z0-9\\s]+$"

description: "Comparators specific to this individual drug component. Can be predefined options or specific drug names. If it is one of the predefined options enumerated in enum but this predefined option is specified (for example, the drug name corresponding to 'best supportive care'), name the specific information as a string, and not as the option from enum."

required: [inn_component, brand_names, individual_comparators]

description: "Individual components of the combination therapy, each with its own INN, brand names, and comparators"

administered_with:

type: [string, "null"]

description: "Name of drug administered with the assessed drug, but not part of the assessment itself"

combination_comparators:

type: array

items:

type: [string, "null"]

oneOf:

- enum: ["standard of care", "best supportive care", placebo, "no treatment", "conventional care", "palliative care", null]

- type: string

description: "Name of drug(s) that this component drug is assessed against"

pattern: "^[a-zA-Z0-9\\s]+$"

description: "Comparators for the combination as a whole, only if this is a combination therapy. Can be predefined options or specific drug names. If it is one of the predefined options enumerated in enum but this predefined option is specified (for example, the drug name corresponding to 'best supportive care'), name the specific information as a string, and not as the option from enum."

outcome_rea:

type: [string, "null"]

enum: [positive, negative, equal, unknown, null]

description: "Outcome of relative effectiveness assessment. This is 'positive' if and only if the drug/combination being assessed is MORE effective than the comparator. Should be 'equal' if the drug/combination being assessed is equally effective as the comparator, or no more effective than the comparator."

outcome_cea:

type: [string, "null"]

enum: [positive, negative, unknown, null]

description: "Outcome of cost-effectiveness assessment for the drug or combination"

final_recommendation:

type: [string, "null"]

enum: [positive, negative, unknown, null]

description: "Final recommendation for the drug or combination"

managed_entry_agreements:

type: [string, "null"]

enum: ["commercial agreement", "outcome-based agreement", null]

description: "Name of managed entry agreement, if any. Only state the name of the managed entry agreement, if any, and no other text."

clinical_restrictions:

type: [string, "null"]

description: "Clinical restrictions, if any."

required: [is_combination_therapy, inn_combination_therapy, components, administered_with, combination_comparators, outcome_rea, outcome_cea, final_recommendation, managed_entry_agreements, clinical_restrictions]

required: [indication_name, technologies]

required: [hta_id, treatment_type, assessment_type, assessment_date, internal_identifier, indications]

**Appendix 4: Examples of useful accuracy.**

|  | Gold standard extraction | Useful accuracy NLP-R |
| --- | --- | --- |
| Example INN | Bevacizumab in combination with gemcitabine and carboplatin | ['bevacizumab', 'gemcitabine', 'platinum', 'carboplatin'] |
| Example indication | advanced (unresectable or metastatic) melanoma in adults after the disease has progressed with ipilimumab and, for BRAF V600 mutation-positive disease, a BRAF or MEK inhibitor | Pembrolizumab is recommended as an option for treating advanced\n(unresectable or metastatic) melanoma in adults only:\n\u2022 after the disease has progressed with ipilimumab and, for BRAF V600\nmutation-positive disease, a BRAF or MEK inhibitor and\n\u2022 when the company provides pembrolizumab in line with the commercial access\nagreement with NHS England |
| Example  Assessment type | Reassessment | ['This guidance replaces TA492.'] |
| Example  Comparator | cytotoxic chemotherapy | [MORE TEXT]…. However, the committee understood that, since 2015,\nretreatment with bortezomib has no longer been available through the Cancer\nDrugs Fund. The committee also heard that NHS England had advised NICE\nthat it would no longer commission retreating multiple myeloma with a\nbortezomib-based therapy. The committee concluded that a bortezomib-\nbased therapy was not an appropriate comparator in this appraisal.\n\u2022 The clinical experts explained that cytotoxic chemotherapy with an alkylating\nagent is a treatment option after bortezomib-based first-line therapies, with an\nalternative alkylating agent taken after disease progression on bortezomib-\nbased therapy. The committee concluded that cytotoxic therapy was a relevant\ncomparator ….[MORE TEXT] |
| Example MEA | Commercial agreement | 1.1 Apalutamide plus androgen deprivation therapy (ADT) is recommended,\nwithin its marketing authorisation, as an option for treating\nhormone-relapsed non-metastatic prostate cancer that is at high risk of\nmetastasising in adults. High risk is defined as a blood prostate-specific\nantigen (PSA) level that has doubled in 10 months or less on continuous\nADT. It is recommended only if the company provides apalutamide\naccording to the commercial arrangement.\nWhy the committee made these recommendations\nHormone-relapsed non-metastatic prostate cancer is usually treated with ADT alone or\nwith darolutamide plus ADT.\nClinical trial evidence suggests that, compared with placebo plus ADT, apalutamide plus\nADT increases the time until the disease spreads and how long people live. The\ncost-effectiveness estimates are within what NICE considers to be an acceptable use of\nNHS resources. So, apalutamide plus ADT is recommended.\nhttps://www.nice.org.uk/guidance/ta740, |

Appendix Table 3: Examples of useful accuracy for the NLP-R method. Abbreviations: managed entry agreement (MEA), natural language processing rule-based (NLP-R).

**Appendix 5 NLP-R extraction analysis on wrong versus missing values.**

| **Attribute** | **Values gold standard** | **Values correct (textually accurate or useful)** | **Values wrong** | **Values missing** |
| --- | --- | --- | --- | --- |
| **Internal identifier** | 50 | 50 | 0 | 0 |
| **HTA ID** | 50 | 50 | 0 | 0 |
| **Brand name** | 50 | 46 | 4 | 0 |
| **INN** | 50 | 45 | 5 | 0 |
| **MAH** | 50 | 46 | 4 | 0 |
| **Assessment type** | 50 | 48 | 2 | 0 |
| **Assessment date** | 50 | 50 | 0 | 0 |
| **Indication** | 50 | 47 | 3* | 0 |
| **Final recommendation** | NA | NA | NA | NA |
| **Comparator** | 50 | 12 | 8 | 30 |
| **Outcome REA** | NA | NA | NA | NA |
| **Outcome CEA** | NA | NA | NA | NA |
| **MEA** | 50 | 33 | 1 | 16 |
| **Clinical restrictions** | NA | NA | NA | NA |

Appendix Table 4: Overview of NLP-R results, including incorrect and missing values. * NLP-R did not find a second indication, so it only returned the first one, classifying the extraction as wrong on a document level. Abbreviations: cost-effectiveness assessment (CEA), health technology assessment organization name (HTA ID), international non-proprietary name (INN), managed entry agreement (MEA), marketing authorization holder (MAH), natural language processing rule-based (NLP-R), relative effectiveness assessment (REA).
